# Drought stress reduces the photosynthetic source of subtending leaves and the transit sink function of podshells, leading to reduced seed weight in soybean plants

**DOI:** 10.3389/fpls.2024.1337544

**Published:** 2024-01-25

**Authors:** Xiangbei Du, Xinyue Zhang, Xiaofei Chen, Wenjun Jin, Zhiping Huang, Lingcong Kong

**Affiliations:** ^1^ Crop Research Institute, Anhui Academy of Agricultural Sciences, Hefei, Anhui, China; ^2^ Jiaxing Academy of Agricultural Sciences, Jiaxing, Zhejiang, China

**Keywords:** subtending leaf, podshell, seed, photoassimilate, soybean

## Abstract

Drought stress is the key factor limiting soybean yield potential. Soybean seed formation involves a coordinated “subtending leaf-podshell-seed” process, but little is known about the assimilation and transport of photoassimilates in subtending leaves, podshells and seeds or their relationships with soybean seed formation under drought stress. To address these research gaps, two-year experiments with two soybean cultivars, Wandou 37 (drought tolerant) and Zhonghuang 13 (drought sensitive), were conducted under three soil water content (SWC) conditions in 2020 and 2021 based on the responses of their yield to drought. We analyzed the photosynthetic assimilation and translocation of photoassimilates in subtending leaves, podshells and seeds by stable isotope labeling. Compared with those under 75% SWC, 60% SWC and 45% SWC significantly decreased the Wandou 37 seed weight by 19.4% and 37.5%, respectively, and that of Zhonghuang 13 by 26.9% and 48.6%, respectively. Compared with those under 75% SWC, drought stress decreased the net photosynthetic rate and the activities of sucrose phosphate synthase (SPS) and sucrose synthase (SuSy), which in turn decreased the photosynthetic capacity of the subtending leaves. The podshells ensure the input of photoassimilates by increasing the SuSy activity, but the weakened source–sink relationship between podshells and seeds under drought stress leads to a decrease in the translocation of assimilates from podshells to seeds. The lack of assimilates under drought stress is an important factor restricting the development of soybean seeds. We conclude that the decrease in seed weight was caused by the decrease in the photosynthetic capacity of the subtending leaves and the decrease in the overall availability of photoassimilates; moreover, by a decrease in the translocation of assimilates from podshells to seeds.

## Introduction

Soybean plants are among the most important oil and industrial crop species worldwide (de Freitas et al., 2019; Pindit et al., 2021), including in China, and are important sources of high-quality protein and vegetable oil (Yang et al., 2016). In China, the Huanghuai area is one of the main soybean producing areas in the country, but droughts often occur from July to August every year, which corresponds to the critical period of soybean seed development, the flowering and pod stage; during this development period, soybean seeds are extremely sensitive to the external environment (Leisner et al., 2017; He et al., 2019). Drought at this time tends to cause an increase in the number of poorly filled pods and a decrease in soybean seed weight. Drought stress is now recognized as a major threat to soybean production in Huanghuai areas and worldwide and can reduce soybean yields by 18.6-83.0% (He et al., 2017; He et al., 2019; Zou et al., 2021). Driven by global climate change, the frequency and intensity of extreme climate events such as droughts are increasing annually (IPCC, 2021).

Photosynthesis is the physiological basis of crop yield, and more than 90% of crop yield is attributed to photoassimilates (Makino, 2011). The photosynthate assimilation and translocation between sources and sinks play critical roles in crop yield formation (Du et al., 2015). The unique growth habit of soybean plants results in the distribution of reproductive organs throughout the plant body; thus, the source–sink relationship in these plants is more complicated than that in other grain crop species (Du et al., 2023). The photoassimilates required for seed development are mainly exported from subtending leaves and subsequently transported to seeds through podshells (Li et al., 2017). Therefore, the soybean seed growth and development processes involve the coordination of three main organs, namely, subtending leaves, podshells and seeds.

Sucrose, the initial product of leaf photosynthesis and the main form of the assimilate that is transported to the seed, is the central hub of carbohydrate metabolism in the soybean source–sink (Du et al., 2020). Sucrose is synthesized in the cytoplasm by enzymes such as sucrose phosphate synthase (SPS) and sucrose synthase (SuSy) (Ishimaru et al., 2008). Studies have shown that the activities of SPS and SuSy in soybean leaves are inhibited under long-term drought stress and that the content of carbohydrates in soybean leaves is consequently reduced, which decreases the synthesis and transport of assimilates (Getachew, 2014). Pods are not only secondary sources of seed development but also transitional sinks through which photoassimilates are translocated from leaves to seeds, and the level of assimilate metabolism strongly influences seed development (Liu et al., 2008; Li et al., 2017). Soybean seeds are considered sinks, the strength of which is determined mainly by the accumulation of assimilates and is affected by the genetic characteristics of the cultivar and by environmental factors, especially water availability (Rotundo and Westgate, 2009; Bellaloui et al., 2015). Seed development also has an important feedback effect on leaf and pod growth, development and photosynthetic energy distribution (Rotundo et al., 2011). The soybean yield is the result of the coordination of subtending leaves, pods and seeds, and failure of one of these organs can lead to yield reduction (Chang and Zhu, 2017; Du et al., 2023). Research on the transport and distribution mechanism of soybean assimilates and their response to drought stress has focused mostly on the roles of single source and sink organs (Bellaloui et al., 2015; He et al., 2017; Leisner et al., 2017; He et al., 2019). However, due to the limitations of research methods and technologies, relatively few studies have investigated the mechanism of the coordinated response of multiple organs to drought stress based on the “subtending leaf-podshell-seed” system.

Our previous research revealed that drought stress significantly reduces photosynthate production in subtending leaves, resulting in a decrease in pod and seed weights (Du et al., 2023). However, the physiological mechanism underlying the effect of drought stress on subtending leaf photosynthate production, the changes in the source–sink relationships of photoassimilates in subtending leaves, podshells and seeds, and the main reasons for the decrease in soybean seed weight under drought stress have not been determined. We speculated that the reduced seed weight of soybean plants under drought stress was associated with subtending leaf photosynthate production and the source–sink relationships of photoassimilates in subtending leaves, podshells and seeds. Therefore, in this study, the effects of drought stress on the assimilation and distribution of photoassimilates via subtending leaf (source)-podshell (transfer sink)-seed (sink) interactions were systematically studied using isotopic labelling technology. The objective of the present study was to investigate the effects of drought stress on the assimilation and translocation of photoassimilates in subtending leaves, podshells and seeds and their relationships with soybean seed formation. The findings of this study are expected to provide insights into the physiological mechanisms of soybean yield formation under drought conditions and provide a new direction for exploring strategies to regulate the soybean response to adverse conditions.

## Materials and methods

### Experimental design

A pilot pot experiment was conducted in Hefei, Anhui, China (31°89′ N, 117°25′ E), in 2019 to test the most drought tolerant and most susceptible soybean cultivars. We evaluated and compared the drought resistance of 21 soybean (*Glycine max* (L.) Merrill) cultivars (Hedou 33, Wanhua 518, Zheng 1307, Shangning 24, Shengdou 101, Wansu 1019, Handou 15, Zhonghuang 302, Shangdou 161, Zheng 1311, Wandou 37, Qihuang 34, Zhonghuang 13, Wandou 39, Zhonghuang 39, Shangdou 1310, Wansu 1208, Zhonghuang 40, Shengdou 5, Jidou 17 and Fudou 18) commonly grown in the Huanghuai area. A two-year (2020-2021) pot experiment was subsequently conducted with the two cultivars, Wandou 37 and Zhonghuang 13, which were identified as the most drought tolerant and the most sensitive cultivar, respectively.

The pot experiments were conducted in a semi-open greenhouse with a transparent waterproof cover that could be rolled up when not used to exclude precipitation. The soybean cultivars were planted in pots (55 cm in height and 60 cm in diameter) that were filled with 30 kg of thoroughly mixed yellow brown loam soil. The soybean cultivars were sown on 10 June 2019, 9 June 2020 and 11 June 2021. Five seeds were sown into each pot, and at the cotyledon stage, the resulting plants were culled to two plants per pot. Approximately 60 pots of each cultivar were planted in 2019, and 300 pots of each cultivar were planted in 2020 and 2021. A uniform fertilizer consisting of 0.5 g N pot^-1^ (60% before sowing and 40% at the flowering stage), 0.5 g P_2_O_5_ pot^-1^ (100% before sowing), and 0.5 g K_2_O pot^-1^ was applied.

All the pots were well watered to maintain a soil water content (SWC) of 75% before the water regimes were established. Drought treatments were established at the R4 stage (there were 2-cm-long pods at each of the 4 nodes in the uppermost part of the main stem with fully grown leaves), and maintained for 30 days. Two water regimes were imposed: 75% SWC and 55% SWC in 2019 and three water regimes were imposed: 75% SWC, 60% SWC and 45% SWC in 2020 and 2021. The error in the SWC was maintained within ±5%. The SWC was measured every 2 days using a soil moisture metre (Spectrum TDR 150, USA) and quantified based on the weighting method of alcohol combustion described by Buske et al. (2013). Every pot was weighted at 17:00-18:00, and daily water consumption was compensated to maintain the SWC. At harvest, all the soybean pods were collected from each plant for measurements of seed yield.

### Sampling and measurements

Flowers at nodes 5 to 8 of the plants were tagged for sampling to determine the flowering date with the aim of ensuring that the labelled flowers had equivalent metabolic and developmental ages. The net photosynthetic rate (*Pn*) on the subtending leaves of the tagged pods was measured using a Li-6400 photosynthetic system (LI-COR Biosciences, Lincoln, NE, USA) under a light intensity of 1500 μmol m^-2^s^-1^ between 9:00 a.m. and 11:00 a.m. every 7 days beginning 5 days after the start of drought treatment (DAT) until pod maturity. The tagged pods and their subtending leaves were sampled every 7 days beginning at 5 DAT until pod maturity. The pods were separated into seeds and podshells, and the leaves were washed with distilled water. One half of the seeds, podshells and leaves were dry-heated at 105°C for 30 min and then at 70°C to a constant weight for measuring the contents of starch and sucrose, and the remainder of the seeds, podshells and leaves were immediately placed in liquid nitrogen and stored at -80°C in an ultralow temperature freezer for measurements of physiological indices. Four replicates of each treatment were included.

### Measurements of the sucrose and starch contents

The sucrose and starch contents of the seeds, podshells and leaves were measured according to the methods described by Chen et al. (2019). The sucrose solution was extracted with 80% ethanol (v/v) and analyzed via an UltiMate 3000 UHPLC (Thermo Fisher Scientific, Waltham, MA, USA) coupled with an ELSD 6000 (Alltech, Deerfield, IL, USA) instrument equipped with a Shodex Asahipak NH_2_P-50 4E column. The residue was used to extract starch with 9.2 mol L^–1^ HClO_4_. The starch concentrations were determined using the anthrone method (Gao, 2006).

### Measurement of enzymatic activity

The activities of SuSy and SPS were measured using biochemical kits (Sucrose synthase test kit and sucrose phosphate synthase test kit) from Suzhou Comin Biotechnology Co., Ltd. (Suzhou, China; http://www.cominbio.com/). The samples were measured following the manufacturer’s instructions.

### ^13^CO_2_ isotopic labelling

To investigate the photosynthetic capacity of the plants and the translocation of photoassimilates from the subtending leaves to the soybean seeds, ^13^CO_2_ feeding was conducted at 11 DAT and 32 DAT in the 2020 and 2021 growing seasons. As the main sources of photosynthetic products to pods, the subtending leaves of pods at the 5th to 8th nodes of the plant were tagged on four different plants from each treatment. The subtending leaves were placed in a transparent and sealed plastic bag between 08:30 a.m. and 9:30 a.m. on the day before sampling, and 5 mL of ^13^CO_2_ (99.9% atom, Shanghai Engineering Center of Stable Isotopes, China) was injected into the plastic bag through a syringe. The bag was then sealed, and 24 h later, the subtending leaf fed ^13^C and the pod attached to this subtending leaf were sampled for determination of the ^13^C content.

### Carbon isotope analysis

The subtending leaves and the pods labelled with ^13^C were sampled and immediately separated from the soybean seeds, and the plant organs were dry-heated at 105°C for 30 min and then at 70°C until a constant weight was reached. All the samples were ground and subsampled for detection of the ^13^C content by using an isotope ratio mass spectrometer (Finnigan MAT, Bremen, Germany) equipped with a Flash HT Plus Elemental Analyser (He et al., 2022). The ^13^C content was calculated according to the equations reported by Baptist et al. (2009) as follows:


$$\text{Atom}\%=\frac{\text{δ}+1000}{\text{δ}+1000+\frac{1000}{{\text{R}}_{\text{standard}}}}$$

whereby δ is the isotopic signature of CO_2_ in the analysed tissue and whereby R_standard_ is the international standard reference (^13^C/^12^C, PeeDee Belemnite). The percent atom excess was then calculated as the difference in % Atom^13^C between labelled and unlabelled kernels.


$$\Delta \text{Atom}\%=\text{Atom}{\%}_{\text{labeled}}-\text{Atom}{\%}_{\text{unlabeled}}$$

The label-derived ^13^C content per dry weight (DW) (γ^13^C, in mg ^13^C g^-1^ DW) in the subtending leaf, podshell and seed was calculated as follows:


$${\text{γ}}^{13}\text{C}=\Delta \text{Atom}\%\times \%\text{C}$$

The ^13^C allocation proportion (%) was calculated as follows:


$${\;}^{13}\text{C allocation proportion }\%=\frac{{\;}^{13}\text{C content in subtending leaf}/\text{podshell}/\text{seed}}{{\;}^{13}\text{C content in subtending leaf}+\text{podshell}+\text{seed}}$$

### Determination of the soybean pod biomass

Soybean pods were collected on each measurement date and separated into seeds and podshells, and their constant weight was determined after they were dried at 70°C in an oven.

### Statistical analysis

Two-way ANOVA, regression analysis and path analysis were performed using IBM SPSS v. 24.0 statistical analysis software. The differences in the means between the three water treatments were tested based on least significant difference (LSD) *post hoc* analysis at the *P* = 0.05 probability level. The Origin 2019 program was used to construct the figures.

## Results

### Drought resistance of various soybean cultivars

**Figure 1:**
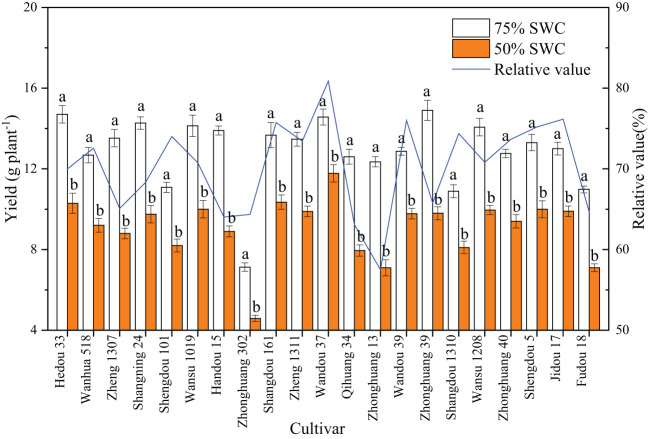
Effects of drought stress on the yield of different cultivars. The data represent the means of four independent replicates ± standard errors (SEs), and different letters on the columns for each cultivar indicate significant differences (*P*< 0.05). SWC, soil water content.

As shown in **Figure 1**, a SWC of 55% significantly decreased the soybean yield relative to that observed with a SWC of 75%. The relative yield was obtained by dividing the yield under 55% SWC by the yield under 75% SWC, and the range of variation between the different cultivars was 57.5-80.9%, with substantial differences among the cultivars. Different cultivars exhibited different degrees of drought tolerance; for example, Wandou 37 was a drought-tolerant cultivar with a relative value of 80.9%, and Zhonghuang 13 was a drought-sensitive cultivar with a relative value of only 57.5%.

### Pod biomass accumulation and partitioning

The pod and seed weights were significantly (*P<* 0.05) affected by the treatment and its interaction with the cultivar (**Table 1**). Compared with those observed in response to a SWC of 75%, the soybean pod and seed weights in response to SWCs of 60% and 45%, were significantly lower, whereas these conditions had no significant impact on the podshell weight of either cultivar. Compared with a SWC of 75%, a SWC of 60% significantly decreased the pod and seed weights of the drought-tolerant cultivar Wandou 37 by an average of 12.4% and 19.4%, respectively, and those of the drought-sensitive cultivar Zhonghuang 13 by an average of 20.0% and 26.9%, respectively. Across both years, a SWC of 45% resulted in 23.9% and 37.5% decreases in the Wandou 37 pod and seed weights, respectively, and 37.5% and 48.6% decreases in the Zhonghuang 13 pod and seed weights, respectively, compared with those observed with a SWC of 75%. No significant difference in the seed weight/pod weight ratio was found between SWCs of 60% and 75%, but a SWC of 45% reduced the seed weight/pod weight ratio for Zhonghuang 13 and Wandou 37.

**Table 1:** Soybean pod biomass production in response to drought stress in 2020 and 2021.

Cultivar	Treatment	2020				2021			
		Pod weight (g)	Seed weight (g)	Podshell weight (g)	Seed weight/pod weight ratio	Pod weight (g)	Seed weight (g)	Podshell weight (g)	Seed weight/pod weight ratio
Wandou 37	75% SWC	0.67 ± 0.02 a	0.48 ± 0.01 a	0.19 ± 0.01 a	0.70 ± 0.02 a	0.75 ± 0.02 a	0.55 ± 0.02 a	0.20 ± 0.01 a	0.76 ± 0.01 a
	60% SWC	0.60 ± 0.02 ab	0.40 ± 0.02 b	0.20 ± 0.01 a	0.66 ± 0.01 ab	0.64 ± 0.01 b	0.43 ± 0.02 b	0.21 ± 0.02 a	0.67 ± 0.02 ab
	45% SWC	0.52 ± 0.02 b	0.31 ± 0.01 c	0.21 ± 0.02 a	0.59 ± 0.02 b	0.56 ± 0.02 c	0.34 ± 0.01 c	0.22 ± 0.01 a	0.60 ± 0.01 b
Zhonghuang 13	75% SWC	0.75 ± 0.02 a	0.52 ± 0.02 a	0.23 ± 0.01 a	0.69 ± 0.03 a	0.83 ± 0.01 a	0.59 ± 0.03 a	0.24 ± 0.02 a	0.71 ± 0.02 a
	60% SWC	0.62 ± 0.01 b	0.40 ± 0.02 b	0.22 ± 0.02 a	0.65 ± 0.02 ab	0.64 ± 0.02 b	0.41 ± 0.02 b	0.23 ± 0.01 a	0.64 ± 0.02 ab
	45% SWC	0.49 ± 0.02 c	0.28 ± 0.01 c	0.21 ± 0.01 a	0.57 ± 0.01 b	0.50 ± 0.02 c	0.29 ± 0.01 c	0.21 ± 0.01 a	0.58 ± 0.01 b
Analysis of variance									
Cultivar (C)		ns	*	*	ns	ns	ns	*	**
Treatment (T)		*	**	ns	ns	**	**	ns	ns
C*T		**	**	ns	ns	**	**	ns	**

Different letters on the column for each cultivar in the same year indicate significant differences (P< 0.05).

SWC, soil water content.

*P< 0.05; **P< 0.01; ns, not significant at P< 0.05.

### Photosynthesis of subtending leaves

**Figure 2:**
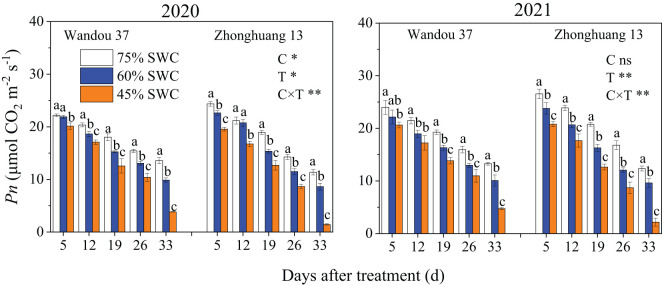
Effects of drought stress on the *Pn* of subtending leaves. The data represent the means of four independent replicates ± standard errors (SEs), and different letters on the columns for each cultivar on the same day indicate significant differences (*P*< 0.05). SWC, soil water content; C, cultivar; T, treatment. *P< 0.05; **P< 0.01; ns, not significant at *P<* 0.05.

The *Pn* of the subtending leaves of both cultivars decreased with time (**Figure 2**), and was significantly affected by treatment and its interaction with the cultivar. The leaf *Pn* significantly decreased in response to SWCs of 60% and 45% compared with that observed with a SWC of 75%. Across both years, SWCs of 60% and 45% resulted in 10.4% and 28.4% decreases, respectively, in the average leaf *Pn* of Wandou 37, and 15.3% and 36.4% decreases, respectively, in the average leaf *Pn* of Zhonghuang 13, compared with those obtained with a SWC of 75%.

### Carbohydrate content and enzyme activities in subtending leaves

**Figure 3:**
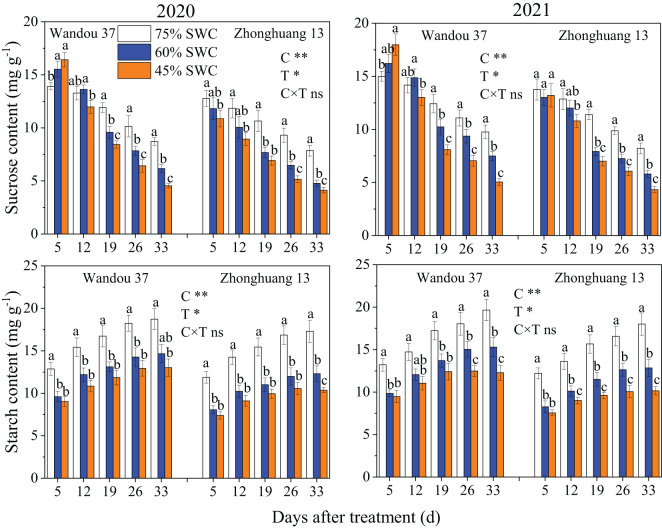
Effects of drought stress on the sucrose and starch contents in subtending leaves. The data represent the mean of four independent replicates ± standard errors (SEs), and different letters on the columns for each cultivar on the same day indicate significant differences (*P*< 0.05). SWC, soil water content; C, cultivar; T, treatment. *P< 0.05; **P< 0.01; ns, not significant at *P<* 0.05.

The sucrose content in the subtending leaves decreased with time in both cultivars, whereas the starch content increased with time (**Figure 3**). The sucrose and starch contents in the subtending leaves were significantly affected by the cultivar and treatment. Compared with the results obtained with a SWC of 75%, the sucrose content of Wandou 37 first significantly increased and then decreased under SWC conditions of 60% and 45%, and that of Zhonghuang 13 decreased under SWC conditions of 60% and 45%. Across both years, SWCs of 60% and 45% resulted in 7.9% and 17.8% decreases, respectively, in the average sucrose contents of Wandou 37, and 20.0% and 28.7% decreases, respectively, in the average sucrose contents of Zhonghuang 13, compared with those obtained with a SWC of 75%. The starch content significantly decreased under SWC conditions of 60% and 45% compared with that obtained with a SWC of 75%. Across both years, SWCs of 60% and 45% resulted in 21.3% and 30.0% decreases, respectively, in the average starch contents of Wandou 37, and 28.2% and 38.2% decreases, respectively, in the average starch contents of Zhonghuang 13, compared with the results obtained with a SWC of 75%.

**Figure 4:**
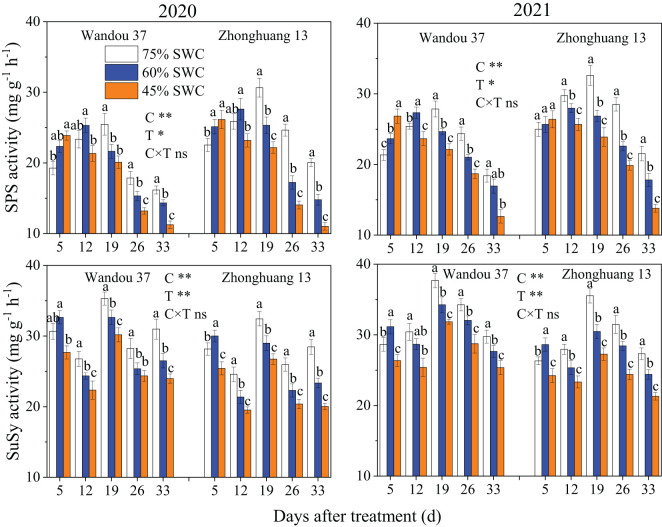
Effects of drought stress on the activities of sucrose phosphate synthase (SPS) and sucrose synthase (SuSy) in subtending leaves. The data represent the means of four independent replicates ± standard errors (SEs), and different letters above the columns for each cultivar on the same day indicate significant differences (*P*< 0.05). SWC, soil water content; C, cultivar; T, treatment. *P< 0.05; **P< 0.01; ns, not significant at *P<* 0.05.

The activity of SPS in subtending leaves exhibited a dynamic trend with a low-high-low single-peak curve with time, and the activity of SuSy in the subtending leaves tended to fluctuate with time (**Figure 4**). The activities of SPS and SuSy in subtending leaves were significantly affected by the cultivar and treatment. In both cultivars, compared with that under a SWC of 75%, the activity of SPS in response to 60% and 45% SWCs first significantly increased and then decreased. Across both years, compared with a SWC of 75%, SWCs of 60% and 45% reduced the activity of SPS in Wandou 37 by 3.2% and 11.7%, and that in Zhonghuang 13 by 11.5% and 21.0%, respectively. In addition, the activity of SuSy under SWCs of 60% and 45% first significantly increased and then significantly decreased, in both cultivars compared with the results obtained under a SWC of 75%. In both years, SWCs of 60% and 45% reduced the activity of SuSy in Wandou 37 by 5.6% and 14.9%, and that in Zhonghuang 13 by 8.7% and 19.3%, respectively, compared with the results obtained with a SWC of 75%.

### Carbohydrate content and enzyme activities in the podshells

**Figure 5:**
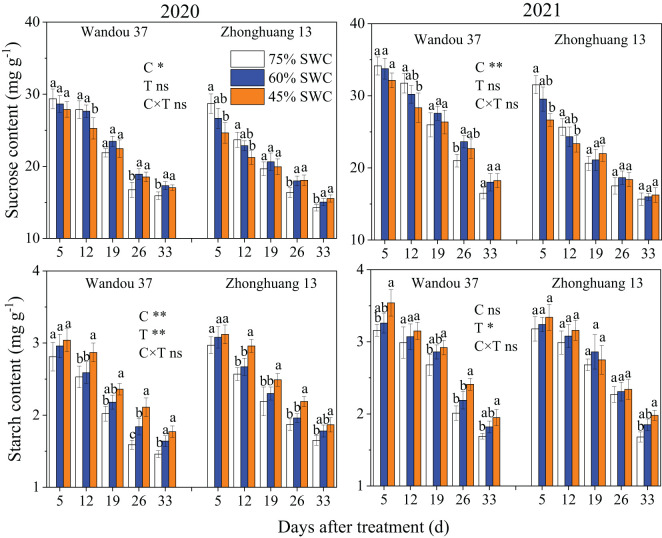
Effects of drought stress on the sucrose and starch contents in the podshells. The data represent the means of four independent replicates ± standard errors (SEs), and different letters above the columns for each cultivar on the same day indicate significant differences (*P*< 0.05). SWC, soil water content; C, cultivar; T, treatment. *P< 0.05; **P< 0.01; ns, not significant at *P<* 0.05.

The sucrose content in the podshells was significantly affected by the cultivar, and the starch content in the podshells was significantly affected by the treatment (**Figure 5**). The sucrose and starch contents in the podshells decreased with time in both cultivars. The sucrose content first decreased and then increased under SWCs of 60% and 45% compared with that observed with a SWC of 75%. Across both years, SWCs of 60% and 45% resulted in 3.3% higher and 0.9% lower average sucrose contents in Wandou 37, respectively, and 0.4% and 3.6% lower average sucrose contents in Zhonghuang 13, respectively, compared with the results obtained with a SWC of 75%. Similarly, the starch content in the podshells under SWC conditions of 60% and 45% were higher than those observed under a SWC of 75%. Moreover, over both years, the average starch content of Wandou 37 and Zhonghuang 13 increased by 6.5% and 14.0%, respectively, under a SWC of 60%, and by 4.5% and 8.9%, respectively, under a SWC of 45%, compared with the results obtained with a SWC of 75%.

**Figure 6:**
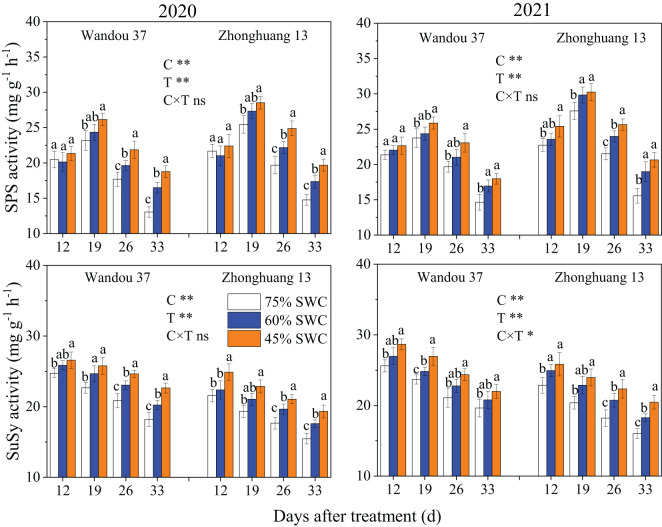
Effects of drought stress on the activities of sucrose phosphate synthase (SPS) and sucrose synthase (SuSy) in the podshells. The data represent the means of four independent replicates ± standard errors (SEs), and different letters above the columns for each cultivar on the same day indicate significant differences (*P<* 0.05). SWC, soil water content; C, cultivar; T, treatment. *P< 0.05; **P< 0.01; ns, not significant at *P<* 0.05.

The activity of SPS in the podshells exhibited a dynamic trend with a low-high-low single-peak curve with time, and the activity of SuSy decreased with time (**Figure 6**). The activities of SPS and SuSy in the podshells were significantly affected by the cultivar and treatment. The activities of SPS and SuSy under SWC conditions of 60% and 45% increase in both cultivars compared with those observed under a SWC of 75%. Across both years, compared with the results obtained with a SWC of 75%, SWCs of 60% and 45% reduced the activity of SPS in Wandou 37 by 7.3% and 15.5%, respectively, and by 9.1% and 16.9% in Zhonghuang 13, respectively, and the activity of SuSy was reduced by 7.2% and 14.2% in Wandou 37, respectively, and by 10.5% and 19.3% in Zhonghuang 13, respectively.

### Carbohydrate content and enzyme activities in seeds

**Figure 7:**
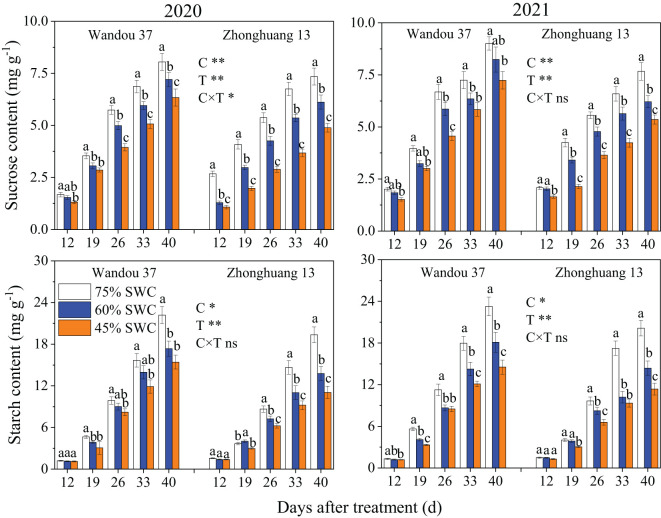
Effects of drought stress on the sucrose and starch contents in the seeds. The data represent the means of four independent replicates ± standard errors (SEs), and different letters above the columns for each cultivar on the same day indicate significant differences (*P*< 0.05). SWC, soil water content; C, cultivar; T, treatment. *P< 0.05; **P< 0.01; ns, not significant at *P<* 0.05.

The sucrose and starch contents in the seeds were significantly affected by the cultivar and treatment (**Figure 7**). The sucrose and starch contents of the seeds increased with time, and significant differences in the changes in the sucrose and starch contents were observed among the treatments. The sucrose and starch contents in both cultivars significantly decreased in response to SWCs of 60% and 45% compared with those observed with a SWC of 75%. In both years, SWCs of 60% and 45% resulted in 11.9% and 23.9% lower average sucrose contents, respectively, in Wandou 37, and 19.7% and 39.8% lower average sucrose contents, respectively, in Zhonghuang 13, compared with those obtained with a SWC of 75%. Moreover, over the two years, the starch content showed average decreases of 19.0% and 29.8% in Wandou 37 and 24.8% and 37.9% in Zhonghuang 13 under SWC conditions of 60% and 45%, respectively, compared with those obtained with a SWC of 75%.

**Figure 8:**
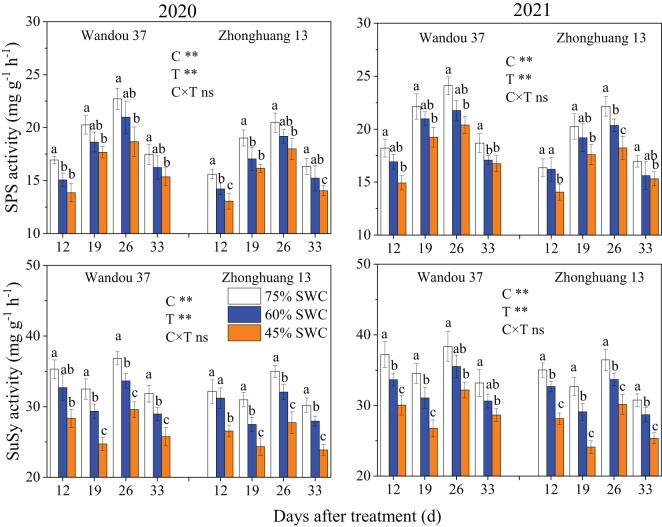
Effects of drought stress on the activities of sucrose phosphate synthase (SPS) and sucrose synthase (SuSy) in the seeds. The data represent the means of four independent replicates ± standard errors (SEs), and different letters above the columns for each cultivar on the same day indicate significant differences (*P<* 0.05). SWC, soil water content; C, cultivar; T, treatment. *P< 0.05; **P< 0.01; ns, not significant at *P<* 0.05.

The SPS and SuSy activities in the seeds were significantly affected by the cultivar and treatment (**Figure 8**). The activity of SPS in the seeds exhibited a dynamic trend with a low-high-low single-peak curve with time, and the activity of SuSy tended to fluctuate with time. The activities of SPS and SuSy under SWCs of 60% and 45% were significantly lower in both cultivars than under a SWC of 75%. Across both years, compared with the results obtained with a SWC of 75%, SWCs of 60% and 45% reduced the activity of SPS by 8.0% and 14.7% in Wandou 37 and by 6.9% and 14.1% in Zhonghuang 13, respectively, and reduced the activity of SuSy by 8.7% and 19.2% in Wandou 37 and by 7.7% and 20.1% in Zhonghuang 13, respectively.

### ^13^C proportions in soybean organs

**Figure 9:**
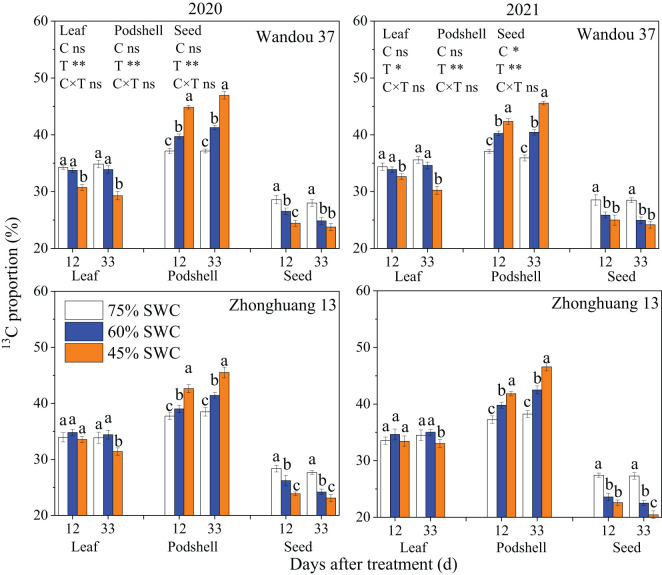
Effects of drought stress on the ^13^C proportion (%) in subtending leaves, podshells and seeds. The data represent the means of four independent replicates ± standard errors (SEs), and different letters above the columns for each cultivar on the same day indicate significant differences (*P*< 0.05). SWC, soil water content; C, cultivar; T, treatment. *P< 0.05; **P< 0.01; ns, not significant at *P<* 0.05.

Our analysis of ^13^CO_2_ in subtending leaves showed that the ^13^C proportions (%) in subtending leaves, podshells and seeds were significantly affected by the treatment. The proportion of ^13^C in the seeds significantly decreased under SWCs of 60% and 45% relative to that found under a SWC of 75% at 12 and 33 DAT in both cultivars, but the proportion of ^13^C in the podshells significantly increased under SWCs of 60% and 45% relative to that under a SWC of 75% at 12 and 33 DAT in both cultivars. Little difference was found in the proportion of ^13^C in the leaves of Wandou 37 and Zhonghuang 13 plants under the different treatments, with the exception that significant decreases in the proportion of ^13^C in the leaves under a SWC of 45% relative to that under a SWC of 75% were found in Wandou 37 plants at 12 and 33 DAT and in Zhonghuang 13 plants at 33 DAT (**Figure 9**). Under SWCs of 60% and 45%, the proportion of ^13^C in the podshells was increased by 7.7% and 17.4% at 12 DAT and by 11.8% and 26.6% at 33 DAT, respectively, in Wandou 37, and by 5.1% and 12.6% at 12 DAT and by 9.4% and 20.0% at 33 DAT, respectively, in Zhonghuang 13, compared with the results obtained with a SWC of 75% across the two years. In addition, under SWCs of 60% and 45%, the proportion of ^13^C in the seeds was decreased by 8.3% and 13.4% at 12 DAT and 11.8% and 15.1% at 33 DAT, respectively, in Wandou 37, and by 10.7% and 16.8% at 12 DAT and 15.0% and 20.8% at 33 DAT, respectively, in Zhonghuang 13, compared with the results obtained under a SWC of 75% across the two years.

### Relationships between assimilation and distribution photosynthates and seed weight

Relationships between the assimilation and transport of photoassimilates in subtending leaves, podshells and seed weight.

**Figure 10:**
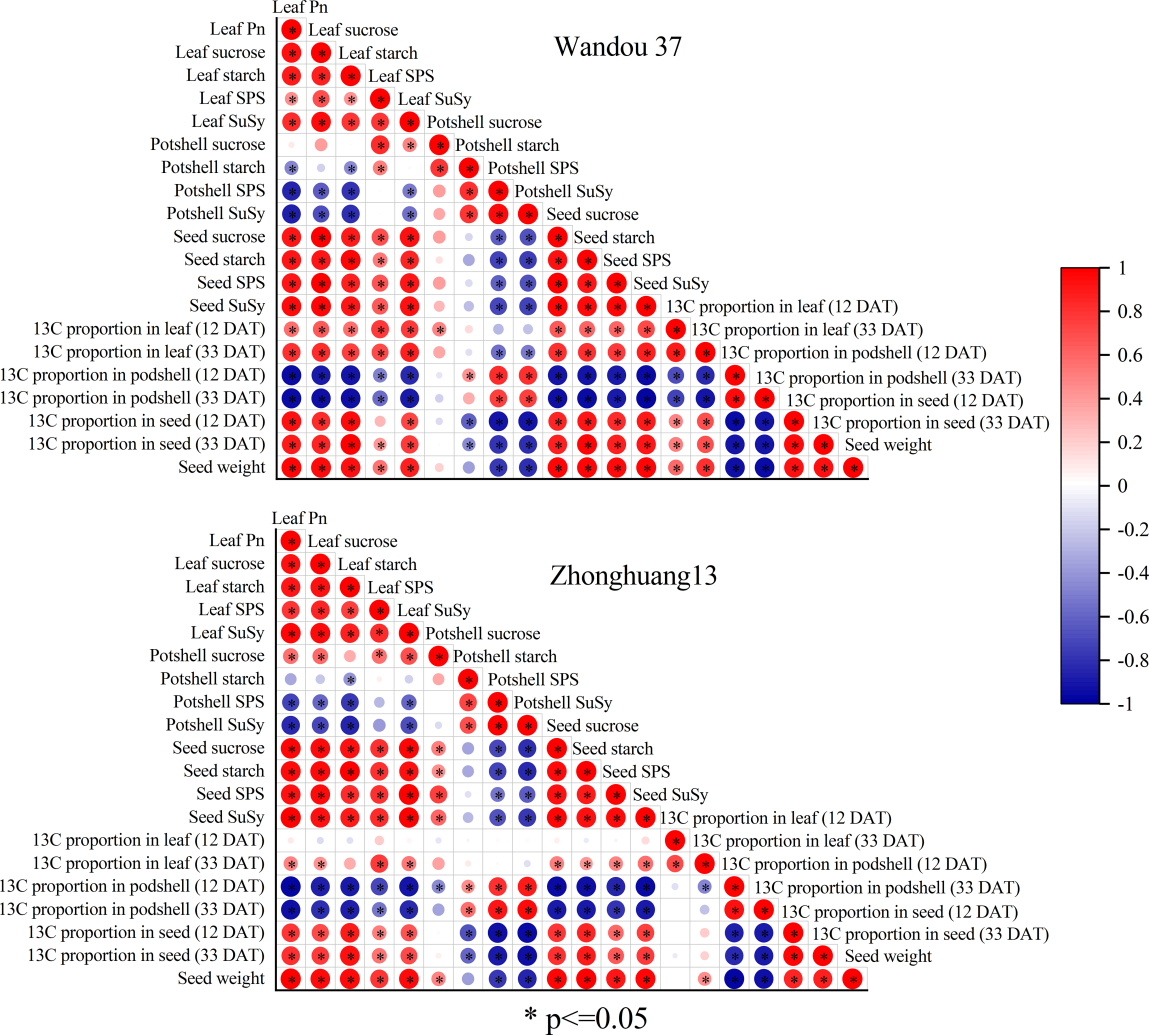
Coefficients of the correlations among the soybean seed weight and physiological indices of subtending leaves, podshells and seeds. SPS, sucrose phosphate synthase; SuSy, sucrose synthase.

Seed weight was positively correlated with leaf *Pn*, leaf sucrose content, leaf starch content, leaf SPS activity, leaf SuSy activity, seed sucrose content, seed starch content, seed SPS activity, seed SuSy activity, leaf ^13^C proportion (12 and 33 DAT) and seed ^13^C proportion (12 and 33 DAT) and negatively correlated with the podshell starch content, podshell SPS activity, podshell SuSy activity and podshell ^13^C proportion (12 and 33 DAT) in Wandou 37. Additionally, the seed weight was positively correlated with the leaf *Pn*, leaf sucrose content, leaf starch content, leaf SPS activity, leaf SuSy activity, podshell sucrose content, seed sucrose activity, seed starch content, seed SPS activity, seed SuSy activity and seed ^13^C proportion (12 and 33 DAT) and negatively correlated with the podshell SPS activity, podshell SuSy activity and podshell ^13^C proportion (12 and 33 DAT) for Zhonghuang 13 (**Figure 10**).

### Stepwise multiple regression analysis

The effects of various physiological indices of different organs on the soybean seed weight could be further explained by a stepwise regression equation (**Table 2**). The effects of the leaf starch content, podshell SPS activity, seed starch content, seed SuSy activity and leaf ^13^C proportion (12 DAT) on seed weight were significant for Wandou 37, and the effects of the seed starch content and seed SuSy activity on seed weight were positive, whereas the effects of the leaf starch content, podshell SPS content and leaf ^13^C proportion (12 DAT) were negative. Moreover, the effects of the leaf *Pn*, leaf sucrose content, leaf starch content, seed SuSy activity, and podshell ^13^C proportion (12 DAT) on the seed weight were significant for Zhonghuang 13, and the effects of the leaf *Pn*, leaf starch content and seed SuSy activity on seed weight were positive, whereas the effects of the leaf sucrose content and the podshell ^13^C proportion (12 DAT) were negative.

**Table 2:** Regression analysis of the soybean seed weight and physiological indices of subtending leaves, podshells and seeds.

Cultivar	Major affecting factors	Regression equation	R^2^
Wandou 37	Leaf starch, Podshell SPS, Seed starch, Seed SuSy, Leaf ^13^C proportion (12 DAT)	Y=0.01553-0.01449Leaf starch-0.00518Podshell SPS+0.03713Seed starch+0.02157Seed SuSy-0.00978Leaf ^13^C proportion (12 DAT)	0.9980^**^
Zhonghuang 13	Leaf *Pn*, Leaf sucrose, Leaf starch, Seed starch, Podshell ^13^C proportion (12 DAT)	Y=0.10998 + 0.01786Leaf *Pn*-0.00438Leaf sucrose+0.00614Leaf starch+0.02463Seed SuSy-0.00516Podshell ^13^C proportion (12 DAT)	0.9993^**^

SPS, sucrose phosphate synthase; SuSy, sucrose synthase.

**, significant at p≤0.01 probability levels.

### Path analysis

The direct diameter coefficient data indicated that the leaf starch content, seed starch content, seed SuSy activity and leaf 13C proportion (12 DAT) had direct negative effects on the seed weight of Wandou 37 plants under drought stress, and the direct path coefficients were -7.798, -3.519, -4.318 and -0.508, respectively (**Table 3**). An analysis of the indirect path coefficients revealed that the seed starch content and seed SuSy activity had stronger indirect effects on the seed weight. In addition, the podshell SPS activity and leaf ^13^C proportion (12 DAT) exerted direct positive effects on the soybean seed weight. In Zhonghuang 13, the leaf *Pn*, leaf sucrose content, leaf starch content and podshell ^13^C proportion (12 DAT) had direct negative effects on the soybean seed weight; among these factors, the leaf *Pn* had the greatest direct effect on the seed weight under drought stress. Seed starch had a direct positive effect on the soybean seed weight. By analyzing the indirect path coefficients, we found that the leaf *Pn* and leaf sucrose content had the strongest indirect effects on seed weight through effects on the seed starch content and the podshell ^13^C proportion (12 DAT).

**Table 3:** Path coefficients of physiological indices of subtending leaves, podshells and soybean seed weight.

Cultivar	Independent variable	Direct path coefficient	Indirect path coefficient					
			Leaf starch	Podshell SPS	Seed starch	Seed SuSy	Leaf 13C proportion (12 DAT)	Total
Wandou 37	Leaf starch	-7.798	—	-1.396	-3.422	-3.949	-0.288	-9.055
	Podshell SPS	1.761	6.179	—	2.543	3.091	0.138	11.951
	Seed starch	-3.519	-7.581	-1.272	—	-4.066	-0.312	-13.231
	Seed SuSy	-4.318	-7.131	-1.261	-3.314	—	-0.363	-12.069
	Leaf 13C proportion (12 DAT)	-0.508	-4.422	-0.479	-2.165	-3.087	—	10.153
			Leaf *Pn*	Leaf sucrose	Leaf starch	Seed starch	Podshell ^13^C proportion (12 DAT)	Total
Zhonghuang 13	Leaf *Pn*	-6.914	—	-0.236	-1.481	11.135	2.145	11.563
	Leaf sucrose	-0.255	-0.638	—	-1.529	11.159	1.935	10.927
	Leaf starch	-1.628	-6.29	-0.24	—	11.407	-1.949	2.928
	Seed starch	11.639	-6.614	-0.245	-1.596	—	2.061	-6.394
	Podshell ^13^C proportion (12 DAT)	-2.218	6.689	0.223	-10.819	-4.57	—	8.477

SPS, sucrose phosphate synthase; SuSy, sucrose synthase.

## Discussion

Compared with a SWC of 75%, drought stress significantly decreased the pod weight by 12.4-23.9% in Wandou 37 and 20.0-37.5% in Zhonghuang 13, and these effects were mainly due to significant decreases in seed weight (**Table 1**). Moreover, the reduction observed with a SWC of 45% was significantly greater than that obtained with a SWC of 60%, and a greater reduction was observed in the drought-sensitive cultivar (Zhonghuang 13) than in the drought-tolerant cultivar (Wandou 37). These results are consistent with previous research results (Ergo et al., 2018; Du et al., 2023). The reason for the decrease in the seed weight is the observed decreases in photosynthetic capacity and the export and transport of photoassimilates in the subtending leaf-podshell-seed system (Griffiths et al., 2016).

Sucrose is the main form of transported photoassimilate and is mainly transported from photosynthetic organs to sink organs over long distances (Ruan, 2012). In this study, we found that the sucrose content in subtending leaves decreased significantly (by 7.9-17.9% and by 20.0-28.7% in Wandou 37 and Zhonghuang 13, respectively) under drought stress (**Figure 3**). Two factors led to the observed decrease in the sucrose content in the subtending leaves. First, the *Pn* decreased in response to drought stress (**Figure 2**). Drought stress inhibits leaf photosynthesis (He et al., 2017; Du et al., 2023), which reduces the formation of photoassimilates and the photosynthetic capacity of leaves. Second, the sucrose synthesis pathway was inhibited by drought stress. The activity of SPS in the subtending leaves decreased significantly under drought stress (**Figure 4**). Because SPS is one of the key enzymes involved in sucrose synthesis in leaves, drought stress blocks the activity of sucrose synthesis and significantly decreases the sucrose content (Getachew, 2014). A decrease in the sucrose content in source organs is not conducive to sucrose export, which leads to the inability of sink organs to obtain sufficient photoassimilates from source organs (Sevanto, 2018). Our results also revealed that the starch content in the subtending leaves of Wandou 37 and Zhonghuang 13 decreased significantly (by 21.3-30.0% and 28.2-38.2%, respectively) under drought stress (**Figure 3**). The reduction in starch content was due to significant decreases in photosynthetic capacity and SuSy activity under drought stress (**Figure 4**). SuSy mainly decomposes sucrose to provide a substrate for starch synthesis (Coleman et al., 2009). The activity of SuSy in leaves significantly decreased, and the amount of substrate for starch synthesis decreased; thus, the starch content was significantly reduced under drought stress. Path analysis also revealed that the factor associated with the direct negative effects of drought stress on the seed weight of the drought-tolerant cultivar Wandou 37 was the leaf starch content, whereas for the drought-sensitive cultivar Zhonghuang 13, the factors were leaf *Pn* and leaf starch content (**Table 3**). Therefore, we can conclude that the reduced photosynthetic capacity of subtending leaves under drought stress is an important factor limiting soybean seed formation.

The podshell mainly performs the functions of sucrose loading at the leaf source and unloading at the seed sink (Dong, 2012). Although the decrease in the photosynthetic capacity of the subtending leaves led to a reduction in the amount of photoassimilates transported to the podshells, the weight of the podshells barely changed (**Table 1**). These results suggest that the podshell is an organ that is almost impervious to drought stress; this finding is consistent with the results of previous studies showing that the podshell is stable under stress (Turner et al., 2005). In the present study, the increase in SPS enzyme activity in the podshells under drought stress promoted the synthesis of sucrose (**Figure 6**), which is conducive to improving osmotic potential, and thus increasing stress resistance (Gangola and Ramadoss, 2018), which may be the reason for the high resistance of podshells under drought stress. However, sucrose is the main form of assimilate transport, and the transmembrane transport of sucrose is regulated by multiple factors, such as intracellular and extracellular sucrose concentration gradients (Ayre, 2011). Excessive sucrose in the podshells would inhibit the input of photoassimilates in the subtending leaves. We found that SuSy enzyme activity in the podshells increased under drought stress, which promoted the conversion of sucrose to starch and ensured the input of photoassimilates from the subtending leaves to the podshells. A portion of the starch can be broken down into sucrose for seed development or converted to other forms of storage. Further analysis indicated that drought stress increased the proportion of assimilates exported from the subtending leaves. The average proportions of assimilates exported from the subtending leaves of the two soybean cultivars under SWCs of 60% and 45% were 65.4% and 67.9%, respectively, which increased by 0.1% and 3.9%, respectively, compared with the results obtained with a SWC of 75% (**Figure 9**). With increasing drought severity, the translocation rate of assimilates from subtending leaves also increased correspondingly. This founding is consistent with previous studies on cotton (Sun et al., 2011). However, with increasing drought severity, the translocation rate of assimilates from the podshells to the seeds decreased. Under SWCs of 60% and 45%, the average translocation rates of assimilates from the podshells to the seeds were 38.0% and 34.5%, respectively, which were 11.4% and 19.6% lower than those obtained with a SWC of 75%. Taken together, these findings indicated that drought stress weakened the source–sink relationship between podshells and seeds, which reduced the translocation of assimilates from podshells to seeds. The described effect results in relative increases in the fraction of assimilates in the podshells, resulting in an increased proportion of ^13^C in the podshells (**Figure 9**). The translocation of assimilates from podshells to seeds was restricted under drought stress and was not conducive to the formation of soybean seeds. Therefore, the translocation of assimilates from podshells to seeds is an important factor limiting the development of soybean seeds under drought stress. The podshell is not only the “transition reservoir” but also the “transit pump” of seed development.

Sucrose is the main carbon source for the development and storage of soybean seeds, whereas starch is a temporary storage form of carbohydrates and can be broken down into hexose to support the development of soybean seeds when the sucrose content is insufficient at the later stage of seed development (Rotundo et al., 2011). In this study, we found that drought stress significantly decreased the sucrose and starch contents in the seeds (**Figure 7**), which indicated that drought stress reduced the distribution of photoassimilates in the seeds and hindered biomass accumulation. Two factors led to decreases in the sucrose and starch contents in the seeds. First, the photosynthetic capacity of subtending leaves decreased significantly under drought stress, leading to a decreases in the translocation of assimilates from subtending leaves to podshells, and the podshell photoassimilates failed to move effectively to the seeds, resulting in a decrease in the translocation of assimilates from podshells to seeds. Second, the activities of SPS and SuSy in the seeds were significantly reduced under drought stress (**Figure 8**), and the activities of sucrose and starch synthesis were blocked (Getachew, 2014), leading to decreases in the sucrose and starch contents in the soybean seeds. Under drought stress, decreases in sucrose and starch contents are not conducive to soybean seed development or the accumulation of stored substances. Our results also revealed that the seed starch content and seed SuSy activity were the major direct factors affecting the negative regulation of seed weight under drought stress (**Table 3**). Under drought stress, decreases in sucrose and starch contents result in a significantly decreased proportion of ^13^C in the seeds (**Figure 9**) and are not conducive to soybean seed development or the accumulation of stored substances. Eventually, drought stress significantly decreased the seed weight (**Table 1**). Therefore, the lack of assimilates under drought stress is an important factor restricting the development of soybean seeds. Similarly, previous studies on rice have shown that abiotic stress results in a decrease in the proportion of assimilates transported to grains and abnormal sucrose metabolism in grains, leading to the formation of blighted grains (Zhang et al., 2017).

In summary, in addition to the decrease in the photosynthetic capacity of subtending leaves and the decrease in the overall availability of photoassimilates, the decrease of translocation of assimilates from the podshells to the seeds is also an important reason for the decrease in seed weight. Drought stress weakened the source–sink relationship between subtending leaves and podshells, the transit sink function of podshells, and the source–sink relationship between podshells and seeds, leading to a decreased seed weight. The weakening effect under a SWC of 45% was significantly greater than that under a SWC of 60%. At the same drought level, the weakening effect of the drought-sensitive cultivar was more obvious than that of the drought-tolerant cultivar. These results confirm our hypothesis that the reduced seed weight of soybean plants under drought stress was caused by a decrease in the photosynthate assimilation in the subtending leaves and in their translocation into podshells and seeds. These results further confirmed that the variation in sucrose content in source and sink organs is very important for the normal growth and development of soybean seeds (He et al., 2017; Leisner et al., 2017). We also found that the podshells plays an important role in the soybean seed yield formation. In addition, this study analysed the effect of drought stress only from the perspective of a single “subtending leaf-podshell-seed” system. In the future, further consideration of the response to drought stress from the perspective of the whole plant is needed. Moreover, studying the compensatory effect of exogenous sucrose on seed weight under drought stress and the mechanisms underlying sucrose metabolism and signaling pathways between source and sink organs under drought stress through joint omics analysis is necessary (Linh et al., 2020).

## Conclusion

Drought stress significantly reduced the seed weight of the soybean plants, and the variation observed under a SWC of 45% was significantly greater than that detected under a SWC of 60%. The drought-sensitive cultivar exhibited more obvious yield reduction effects than did the drought-tolerant cultivar. The main physiological reasons for the decrease in seed weight were a decrease in the photosynthetic capacity of the subtending leaves, as well as the decrease in the translocation of assimilates from the podshells to the seeds.

## Data availability statement

The raw data supporting the conclusions of this article will be made available by the authors, without undue reservation.

## Author contributions

XD: Conceptualization, Funding acquisition, Investigation, Resources, Writing – original draft. XZ: Investigation, Methodology, Writing – original draft, Writing – review & editing. XC: Data curation, Writing – review & editing. WJ: Investigation, Visualization, Writing – review & editing. ZH: Conceptualization, Investigation, Visualization, Writing – review & editing. LK: Conceptualization, Resources, Visualization, Writing – review & editing.
